# The most influential articles in total elbow arthroplasty: bibliometric analysis of the top 100 most-cited articles

**DOI:** 10.1016/j.xrrt.2026.100821

**Published:** 2026-07-17

**Authors:** Brian S. Tao, Timothy W. Kouo, Hailey B. Hulse, Kevin A. Hao, Antonio Cusano, Robert L. Parisien, Andrew B. Stein, Josef K. Eichinger, Richard Ma, Xinning Li

**Affiliations:** a Boston University School of Medicine, Boston, MA, USA; b Anne Burnett School of Medicine at Texas Christian University, Fort Worth, Texas, USA; c University of Illinois College of Medicine, Rockford, IL, USA; d Department of Orthopaedic Surgery & Sports Medicine, University of Florida, Gainesville, FL, USA; e Department of Orthopaedic Surgery, University of Connecticut, Farmington, CT, USA; f Department of Orthopaedics, Icahn School of Medicine at Mount Sinai, New York, NY, USA; g Medical University of South Carolina, Charleston, SC, USA; h Department of Orthopaedic Surgery, University of Missouri School of Medicine, Columbia, MO, USA

**Keywords:** Total elbow arthroplasty, TEA, Elbow replacement, Bibliometric analysis, Citations, Most cited articles

## Abstract

**Background:**

The literature surrounding total elbow arthroplasty (TEA) has evolved as advances in prosthetic design, surgical technique, and patient selection have shaped its modern role in elbow reconstructive surgery. We hope to identify and examine the characteristics of the 100 most-cited articles on TEA in the orthopedic literature.

**Methods:**

A bibliometric analysis was conducted using the Web of Science databases with the search term: “total” AND “elbow arthroplasty.” Articles were included if the primary focus was on TEA. Studies addressing epicondylitis, elbow stiffness, or general elbow surgery without explicit reference to TEA were excluded. The top 100 most-cited articles were analyzed for publication year, total citations, citations per year, level of evidence, study design, authors, institutions, country of origin, and journal impact.

**Results:**

The top 100 most-cited TEA articles received a total of 8,225 citations, averaging 82 citations per article. Citations have steadily increased since 1990, with the most productive publication year being 2005. The most cited article, by Day et al, examined national trends in upper extremity arthroplasty and projected future growth in procedure volumes. The 3 most cited articles were each cited more than 250 times. The top journals were Journal of Bone and Joint Surgery–American Volume (3,632 citations), Journal of Shoulder and Elbow Surgery (1,550 citations), and Journal of Bone and Joint Surgery–British Volume (753 citations). Dr. Bernard Morrey was the most prolific author with 35 publications and 3,125 citations, followed by Dr. Robert Adams. The United States led in publication volume (n = 58), specifically Mayo Clinic (n = 30). Most studies were level 4 evidence (n = 59). No significant correlation was found between level of evidence and total citations (*P* = .21) or between journal impact factor and citation rate when adjusted for study design and evidence level (*P* = .08). Keyword network analysis identified central themes around TEA, elbow, arthroplasty, arthritis, and revision.

**Conclusion:**

The Day et al study on national trends and projections in upper extremity arthroplasty is the most cited work in the TEA literature. The growing number of citations over time reflects TEA's rising clinical relevance and the evolving landscape of elbow reconstruction. Despite most articles being lower level of evidence, the citation impact surrounding TEA appears more closely tied to its clinical relevance than its methodology.

Total elbow arthroplasty (TEA) is a specialized surgical procedure primarily indicated for patients with end-stage elbow arthritis, complex distal humerus fractures, and certain inflammatory conditions like rheumatoid arthritis.^31^^,^^56^ While less common than hip or knee arthroplasty, TEA has demonstrated significant benefits in pain relief and functional restoration, especially in elderly patients and those with inflammatory arthropathies.^21^^,^^39^ However, the procedure carries a relatively high complication rate compared to other joint arthroplasties.^14^ Common complications include implant loosening, periprosthetic fractures, triceps insufficiency, infection, and ulnar nerve palsy. Despite these risks, many patients achieve satisfactory outcomes, with studies reporting high rates of pain relief and improved range of motion.^21^ Long-term success is closely linked to appropriate patient selection and adherence to post-operative guidelines, such as lifelong restrictions on heavy lifting to minimize implant stress.^20^^,^^23^

The evolution of TEA has been marked by significant advancements since its inception. The first documented elbow joint replacement was performed by Robineau in 1925, using a prosthesis made of metal and vulcanized rubber.^24^^,^^70^ It was not until 1972 that the modern era of TEA began, when Dee introduced a cemented hinged prosthesis that addressed earlier issues of poor fixation and instability. Subsequent developments led to the creation of various implant designs, including linked (semi-constrained) and unlinked (non-constrained) prostheses.^27^^,^^29^ Linked designs offer greater stability and are often used in cases with significant bone loss or ligamentous insufficiency, while unlinked designs aim to preserve more natural joint kinematics but require intact soft tissue structures.^4^ These innovations have expanded the indications for TEA and improved patient outcomes, although challenges such as implant longevity and complication rates continue to drive ongoing research and refinement in prosthetic design and surgical techniques.^13^^,^^30^

Bibliometric studies provide valuable insights into the evolution of research and its impact on clinical practice. While similar bibliometric analyses have been performed in other areas of orthopedic and elbow surgery, no such analysis has been conducted specifically as it pertains to TEA.^3^^,^^8^^,^^18^^,^^65^ The purpose of this study is to perform a bibliometric analysis of the most-cited articles on TEA. By identifying and evaluating these influential studies, we seek to provide a comprehensive overview of the evolution, current understanding, and literature trends related to TEA, thereby informing areas of future investigations and clinical practices.

## Methods

All databases in Web of Science, which includes Web of Science Core Collection, Chinese Science Citation Database, Russian Science Citation Index, Arabic Citation Index, and Book Citation Index, and Scientific Electronic Library Online, were queried on April 15, 2025 using the following Boolean search term: “total” AND “elbow arthroplasty.” Because the purpose of this bibliometric analysis was to identify the most-cited articles whose primary focus was TEA, we selected a focused and reproducible search strategy using the commonly employed term “total elbow arthroplasty” rather than a broader synonym-based string. The Web of Science platform was selected as the citation source for this bibliometric analysis because it provides broad multidisciplinary coverage across multiple citation indexes and hosted collections and is widely used for citation-based research. PubMed itself is not included within Web of Science, although MEDLINE may be searched on the Web of Science platform as a separate hosted collection. We used a single-citation database because citation counts vary across platforms, and use of one internally consistent source improves reproducibility of article ranking and citation quantification. All articles since inception were considered. To improve sensitivity, no filters were added. Articles were included if the primary focus was related to TEA. Articles addressing epicondylitis, elbow stiffness, and surgical management of the elbow without explicit reference to TEA were excluded.

The top 100 most-cited articles were reviewed by the first 3 authors (B.T., T.K., and H.H.) to obtain article information including author names, publication year, country of origin, journal name, total number of citations, and the level of evidence. If there was no consensus on inclusion/exclusion of an article or information extracted from an article, it was left to the discretion of the senior author (X.L.).

Analysis was performed in python 3.10.0 and plots were generated using the matplot and plotly libraries. Network analysis was performed using the network package. Spearman coefficient analysis was used to assess the correlation of a study's level of evidence and the amount of citations received. Multivariable linear regression adjusted for study design and level of evidence was used to identify potential relationship of journal impact factor on an article's citations per year (CPY). Calculations of citation per year (number of citations/year since publication) used 2024 as the most recent year for analysis. Although the database search was performed in April 2025, CPY were standardized to 2024, the last fully completed calendar year, to minimize partial-year bias and avoid disadvantaging more recently published articles. Analysis was also performed on citation density (the number of citations for an article based on the year of publication). Country of origin was determined by the affiliation of the first author for each article. If the level of evidence was not explicitly reported for an article, the Journal of Bone and Joint Surgery (JBJS) guidelines were used.^42^ The impact factor for each journal was gathered from the official Clarivate reports for the most recent year available.^16^ When level of evidence was not explicitly reported, it was independently assigned by the first 2 authors according to JBJS guidelines. Any discrepancies were resolved through discussion and, when needed, adjudication by the senior author.

## Results

The top 100 most-cited TEA articles received 8,225 citations with an average 82 ± 69 citations per article and a range of 34 to 530 citations (Appendix 1). In terms of CPY, the top 100 articles had an average of 5 ± 4 CPY, with a minimum of 1.3 CPY and a maximum of 35.3 CPY. Of note, the top 7 articles were each cited more than 200 times.

### Characteristics of the top 10 most-cited total elbow arthroplasty articles

The most cited article on TEA (n = 530) is a database study published by Day et al in 2010 in the Journal of Shoulder and Elbow Surgery.^22^ This study analyzed national trends in shoulder and elbow arthroplasty in the United States from 1993 to 2007 and projected future growth through 2015 using large-scale inpatient and census data. It found that procedure volumes for upper extremity arthroplasty were increasing rapidly, at rates comparable to or exceeding those of hip and knee replacements, with projected increases of up to 322%. Day et al also highlighted a growing revision burden and rising healthcare costs associated with these procedures. This study was one of the first large-scale projections to underscore the accelerating demand for shoulder and elbow arthroplasty and the potential implications for resource allocation and healthcare planning. The second most cited article (n = 289), authored by McKee et al and published in 2009 also in the Journal of Shoulder and Elbow surgery, was a multicenter randomized controlled trial that compared TEA and open reduction and internal fixation (ORIF) in elderly patients with displaced intra-articular distal humerus fractures, finding that TEA provided significantly better functional outcomes and more predictable recovery over 2 years.^43^ TEA also showed shorter operative times, better early Disabilities of the Arm, Shoulder and Hand scores, and a trend toward fewer reoperations, especially in cases where fixation was not feasible due to comminution.

McKee et al’s article established high-level evidence that supports TEA as a preferred primary treatment option in selected elderly patients with complex distal humeral fractures, shifting the paradigm away from traditional ORIF and influencing clinical decision-making. The third most cited article (n = 271), a long-term follow-up study by Gill et al published in 1998 in JBJS-American Volume, evaluated the outcomes of Coonrad Morrey TEA in patients with rheumatoid arthritis, demonstrating a 92.4% prosthesis survival rate at 10-15 years and high levels of patient satisfaction.^28^ Most patients experienced pain relief, improved range of motion, and functional gains, with a relatively low rate of serious complications or revisions. This was some of the earliest and most robust long-term data supporting TEA as a durable and effective treatment for rheumatoid arthritis‑related elbow destruction, helping to establish it as the gold standard in this population. The research topic and results of the top 10 most-cited TEA articles are presented in Table I. The top 10 TEA articles by CPY are reported in Table II.

**Table I:** Top 10 most-cited total elbow arthroplasty articles

Rank	Title	Authors	Citations	Summary
1	Prevalence and projections of total shoulder and elbow arthroplasty in the United States to 2015	Day, Judd S.	530	A retrospective epidemiological study analyzing US trends in shoulder and elbow arthroplasty from 1993 to 2007 projected a 192% to 322% increase in procedures by 2015, with annual growth rates between 6% and 13%. The study also noted a rise in the revision burden from 4.5% to 7%, alongside increased hospital charges, highlighting potential financial implications for the healthcare system.
2	A multicenter, prospective, randomized, controlled trial of open reduction-internal fixation versus total elbow arthroplasty for displaced intra-articular distal humeral fractures in elderly patients	McKee, Michael D.	289	A multicenter randomized controlled trial comparing open reduction–internal fixation and total elbow arthroplasty for displaced intra-articular distal humeral fractures in elderly patients found that TEA resulted in significantly better functional outcomes, as measured by the Mayo Elbow Performance Score at multiple post-operative intervals up to 2 yr. Although early DASH scores favored TEA, differences diminished over time, and reoperation rates were not statistically different between the groups.
3	The Coonrad Morrey total elbow arthroplasty in patients who have rheumatoid arthritis - A ten to fifteen-year follow-up study	Gill, D.R.J.	271	A long-term cohort study evaluated outcomes of the Coonrad Morrey total elbow arthroplasty in 78 elbows from 69 patients with rheumatoid arthritis and found a 92.4% prosthesis survival rate at 10-15 yr. Most patients reported minimal or no pain, significant improvement in range of motion, and 86% had good or excellent outcomes by Mayo Elbow Performance Score, with a 14% overall complication rate.
4	Total elbow arthroplasty as primary treatment for distal humeral fractures in elderly patients	Cobb, T.K.	255	A retrospective case series evaluated primary total elbow arthroplasty for acute distal humerus fractures in 21 elbows from 20 patients, primarily elderly, and found excellent or good functional outcomes in all but one case. With a mean follow-up of 3.3 yr, the study reported high implant survival, improved range of motion, and minimal complications, supporting TEA as a viable alternative to fixation in elderly patients with complex fractures.
5	TOTAL ELBOW ARTHROPLASTY - A 5-YEAR EXPERIENCE AT THE MAYO-CLINIC	Morrey, B.F.	246	A comparative study of 80 total elbow arthroplasties performed at the Mayo Clinic over 5 yr found good outcomes in 60% of cases, with significant improvements in pain relief and range of motion. Despite a high complication rate, particularly in the early years, successful cases showed excellent stability and functional recovery.
6	A comparison of open reduction and internal fixation and primary total elbow arthroplasty in the treatment of intra-articular distal humerus fractures in women older than age 65	Frankle, M.A.	228	A retrospective comparative study evaluated ORIF vs. total elbow arthroplasty in women aged more than 65 yr with intra-articular distal humerus fractures and found superior outcomes in the TEA group, with 11 of 12 patients achieving excellent results and no need for revision. In contrast, the ORIF group had a higher rate of poor outcomes and revisions, supporting TEA as a strong treatment option in this population.
7	EXTENSIVE POSTERIOR EXPOSURE OF THE ELBOW - A TRICEPS-SPARING APPROACH	Bryan, R.S.	225	A technical report described a triceps-sparing posterior approach to the elbow designed to reduce complications like triceps avulsion following total elbow arthroplasty. By reflecting the triceps mechanism in continuity with surrounding soft tissues, this technique provided excellent joint exposure without compromising triceps function and was successfully used in 49 consecutive cases.
8	Functional outcome of semiconstrained total elbow arthroplasty	Hildebrand, K.A.	158	A prospective outcomes study assessed semi-constrained total elbow arthroplasty using the Coonrad Morrey prosthesis and found significantly better functional scores in patients with inflammatory arthritis compared to those with traumatic or post-traumatic conditions. Despite high patient satisfaction overall, the procedure was associated with notable complication rates, including ulnar nerve dysfunction, intraoperative fractures, and triceps disruption.
9	Distal humeral fractures treated with noncustom total elbow replacement	Kamineni, S.	149	A retrospective review of 49 distal humeral fractures treated with non-custom total elbow arthroplasty found high functional scores and reliable outcomes in elderly patients, with an average Mayo Elbow Performance Score of 93 at follow-up. While 29% experienced a complication, most did not require additional surgery, supporting TEA as a dependable option when reconstruction is not feasible.
10	Complex fractures of the distal humerus in the elderly - The role of total elbow replacement as primary treatment	Garcia, J.A.	146	A retrospective study of 19 elderly patients with complex distal humerus fractures treated with primary total elbow arthroplasty showed excellent functional outcomes, with a mean Mayo Elbow Performance Score of 93 and high patient satisfaction. Pain levels were minimal, stability was maintained, and complications were rare, supporting TEA as a reliable option when reconstruction is not feasible.

*TEA*, total elbow arthroplasty; *DASH*, Disabilities of the Arm, Shoulder and Hand; *ORIF*, open reduction and internal fixation.

**Table II:** Top 10 most-cited total elbow arthroplasty articles by citations per year

Rank	Title	Authors	CY	Total citations
1	Prevalence and projections of total shoulder and elbow arthroplasty in the United States to 2015	Day, Judd S.; Lau, Edmund; Ong, Kevin L.; Williams, Gerald R.; Ramsey, Matthew L.; Kurtz, Steven M.	35.3	530
2	A multicenter, prospective, randomized, controlled trial of open reduction-internal fixation versus total elbow arthroplasty for displaced intra-articular distal humeral fractures in elderly patients	Mckee, Michael D.; Veillette, Christian J.H.; Hall, Jeremy A.; Schemitsch, Emit H.; Wild, Lisa M.; McCormack, Robert; Perey, Bertrand; Goetz, Thomas; Zomar, Mauri; Moon, Karyn; Mande, Scott; Petit, Shirlet; Guy, Pierre; Leung, Irene	18.1	289
13	Total Elbow Arthroplasty: A Systematic Review.	Welsink, Chantal L; Lambers, Kaj T.A.; van Deurzen, Derek F.P.; Eygendaal, Denise; van den Bekerom, Michel P.J.	14	112
6	A comparison of open reduction and internal fixation and primary total elbow arthroplasty in the treatment of intraarticular distal humerus fractures in women older than age 65	Frankle, M.A.; Herscovici, D; DiPasquale, T.G.; Vasey, M.B.; Sanders, R.W.	10.4	228
3	The Coonrad Morrey total elbow arthroplasty in patients who have rheumatoid arthritis - A ten to fifteen-year follow-up study	Gill, D.R.J.; Morrey, B.F.	10	271
4	Total elbow arthroplasty as primary treatment for distal humeral fractures in elderly patients	Cobb, T.K.; Morrey, B.F.	9.1	255
39	Total Elbow Arthroplasty for Distal Humeral Fractures A Ten-Year-Minimum Follow-up Study	Barco, Raul; Streubel, Philipp N.; Morrey, Bernard F.; Sanchez-Sotelo, Joaquin	8.4	67
16	Indications and Reoperation Rates for Total Elbow Arthroplasty: An Analysis of Trends in New York State	Gay, David M.; Lyman, Stephen; Huong Do; Hotchkiss, Robert N.; Marx, Robert G.; Daluiski, Aaron	8.2	106
26	Total elbow replacement: outcome of 1,146 arthroplasties from the Scottish Arthroplasty Project	Jenkins, Paul J.; Watts, Adam C.; Norwood, Tim; Duckworth, Andrew D.; Rymaszewski, Lech A.; McEachan, Jane E.	7.6	91
50	Why does total elbow arthroplasty fail today? A systematic review of recent literature	Prkic, Ante; Welsink, Chantal; The, Bertram; van den Bekerom, Michel P.J.; Eygendaal, Denise	7.6	61

### Characteristics of the top 100 most-cited total elbow arthroplasty articles

**Figure 1:**
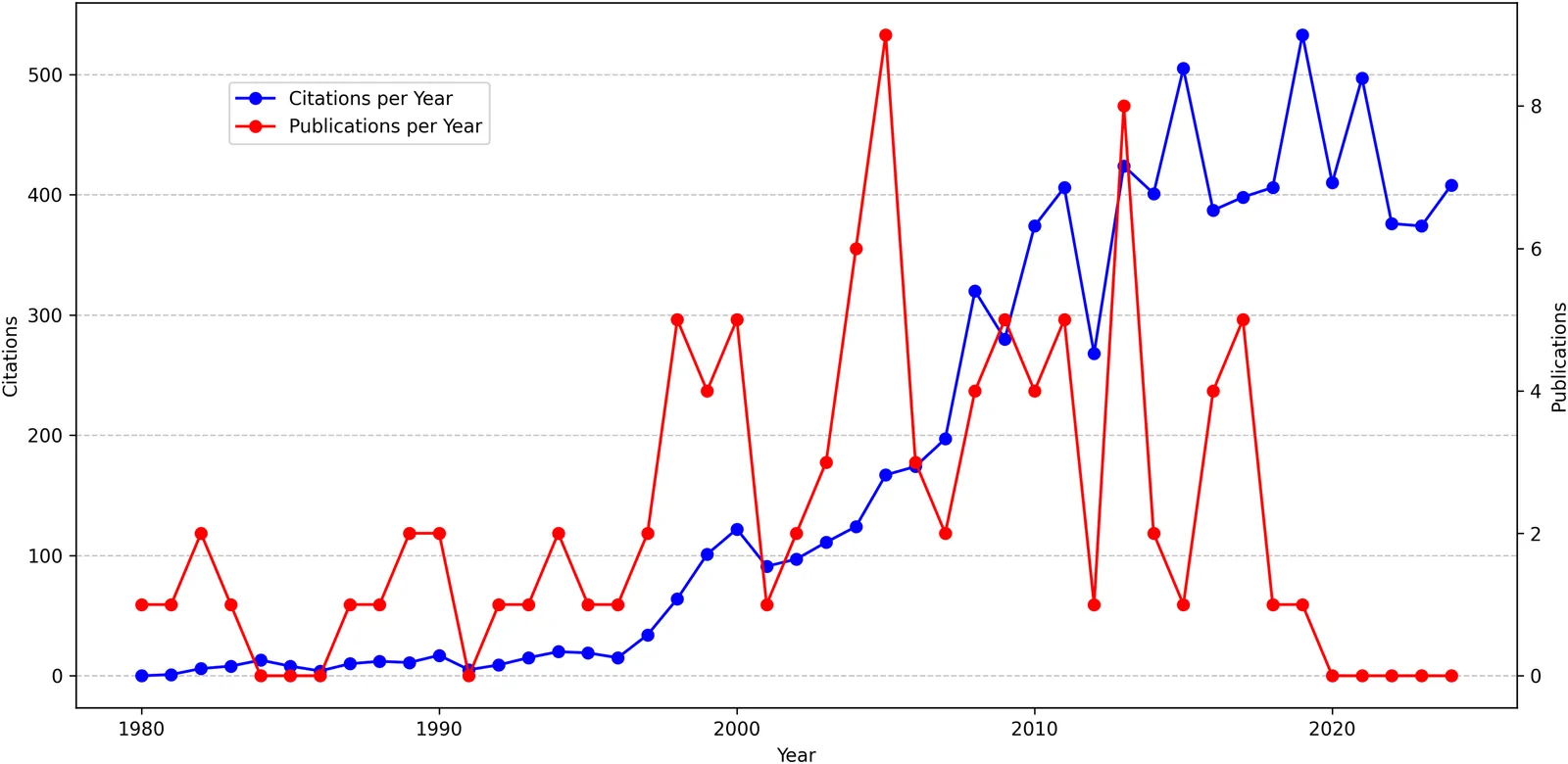
Citations and publications per year for top 100 most-cited total elbow arthroplasty articles.

**Figure 2:**
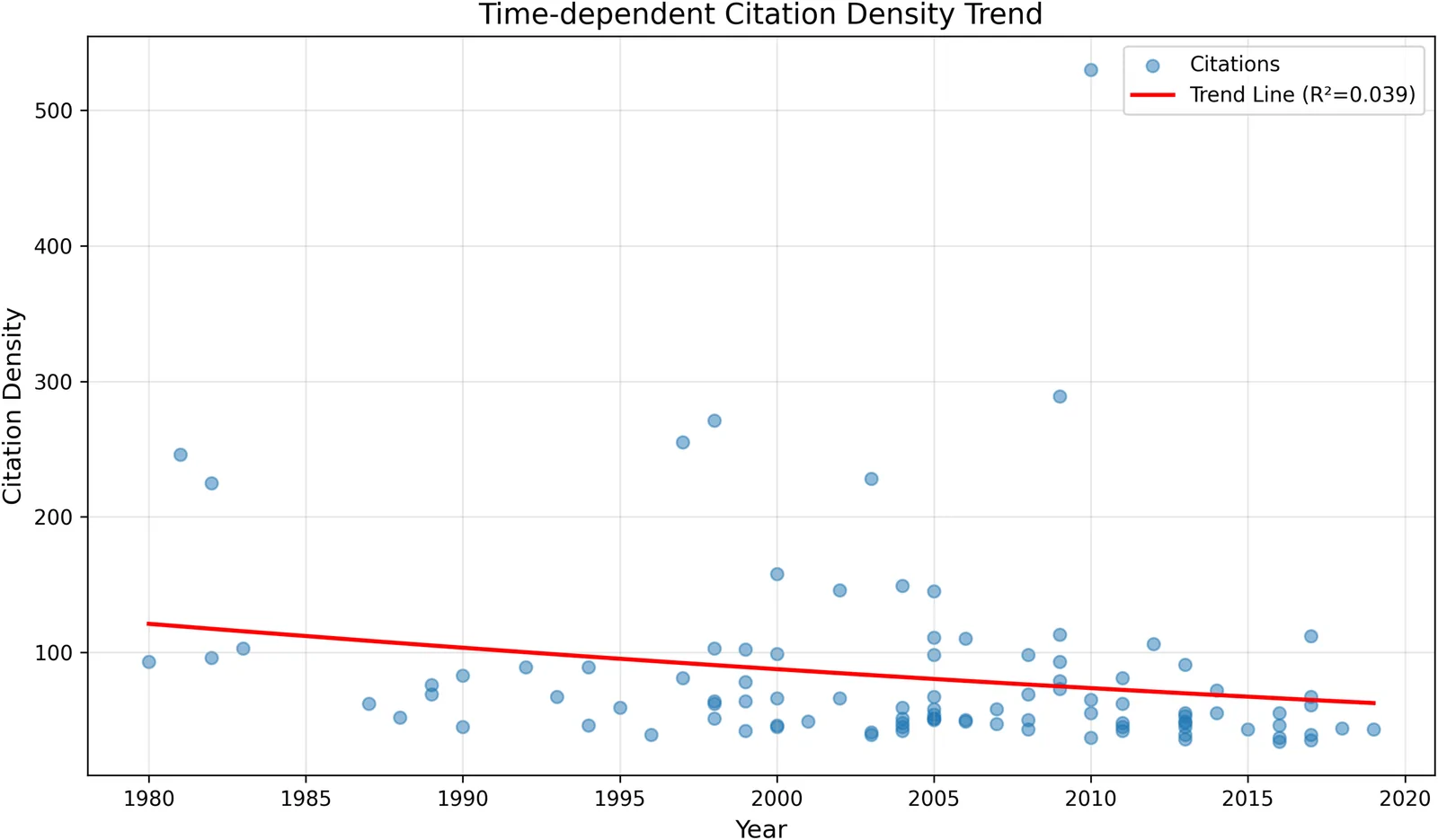
Citation density trend for top 100 most-cited total elbow arthroplasty articles.

Of the top 100 most-cited TEA articles, the year with the greatest number of article publications from that list occurred in 2005 (n = 9; Fig. 1). In terms of citations for TEA, there has been a steady upward trajectory from 1990 until 2015. Of the top 100 most-cited TEA articles from the Web of Science databases, the earliest was Ewald et al in 1980, while the latest was Zhang et al in 2019.^25^^,^^70^ Citation density showcases a weak decline CPY of articles with recency of publication (Fig. 2).

**Figure 3:**
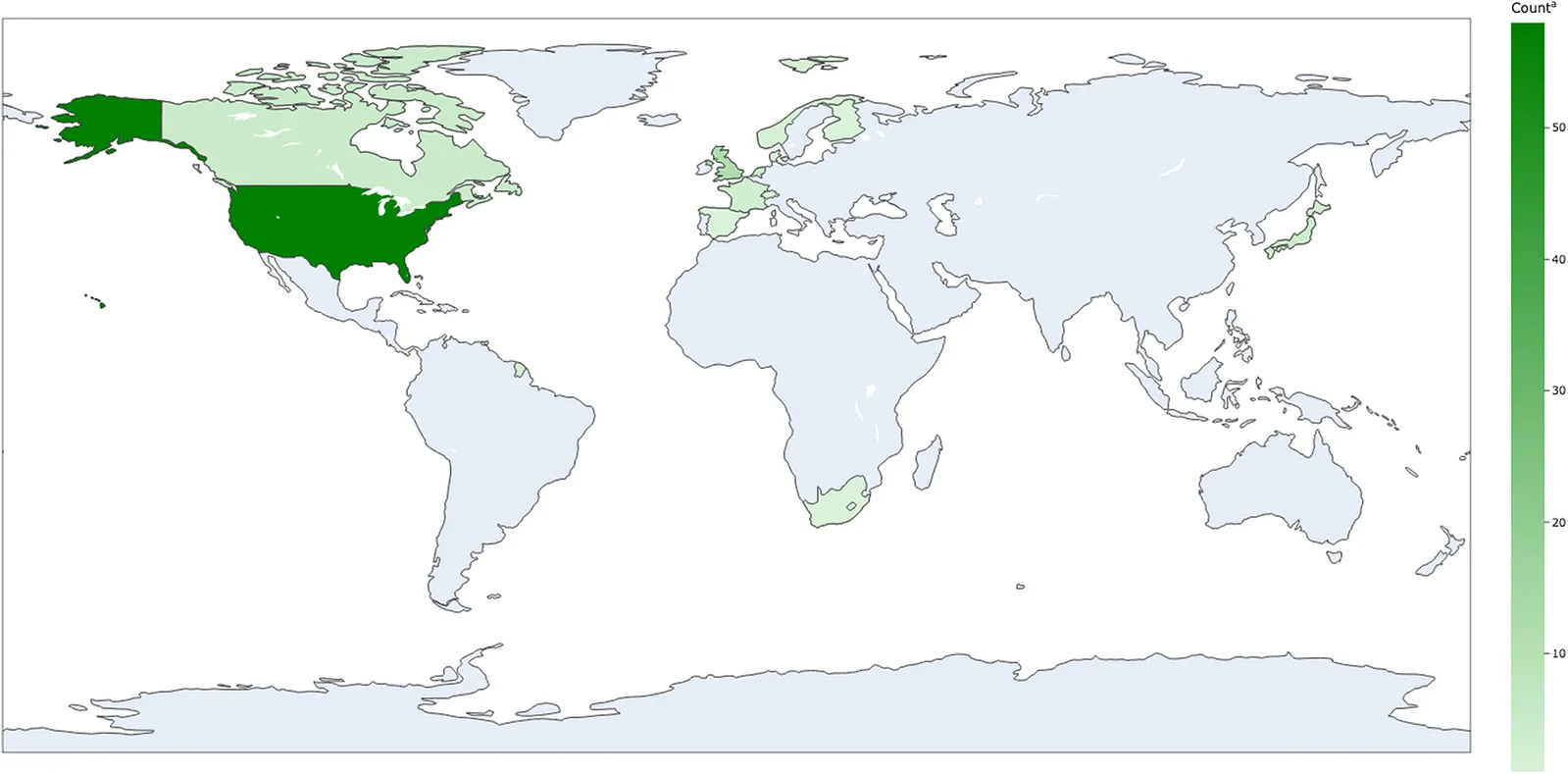
Country distribution for top 100 most-cited total elbow arthroplasty articles.

A total of 21 journals made up the most cited TEA articles. The most cited journals were JBJS–American Volume (citations = 3,632), Journal of Shoulder and Elbow Surgery (citations = 1,550), and JBJS–British Volume (citations = 753). A total breakdown for the top 10 journals can be found in Table III. When adjusted for level of evidence and study design, there was no correlation between journal impact factor and CPY (*P* = .08). The most prolific institution was Mayo Clinic, making up 30% of all top cited TEA articles (Table IV). Dr. Bernard Morrey was the most prolific author with 35 publications in the top 100 TEA articles, also having the most citations at 3,125. Further breakdown for the top 10 prolific authors can be found in Table V. There were 14 senior authors who had at least 2 top cited TEA articles. Of them, Dr. Bernard Morrey also had the most in terms of senior authorship at 29 articles as a senior author (Table VI). The United States had the most TEA articles at 58, while the United Kingdom (n = 12) and the Netherlands (n = 6) had the second and third most cited TEA articles, respectively (Fig. 3). An additional analysis of the most recent articles within the top cited TEA literature demonstrated that recent influential studies have primarily focused on implant survivorship, complication patterns, and outcomes in fracture-related indications, reflecting a shift toward large cohort and systematic review methodologies in contemporary TEA research (Table VII).

**Table III:** Top journals for top 100 most-cited total elbow arthroplasty articles

Journal	Total citations	Count	CY	Impact factor
Journal of Bone and Joint Surgery‑American Volume	3,632	43	162.5	4.4
Journal of Shoulder and Elbow Surgery	1,550	16	112.3	2.9
Journal of Bone and Joint Surgery‑British Volume	753	8	32.3	4.9
Clinical Orthopaedics and Related Research	492	5	13.5	4.4
Journal of Orthopaedic Trauma	324	3	17.2	1.6
Injury–International Journal of the Care of the Injured	172	2	8.5	2.2
ACTA Orthopaedica	170	2	12.5	2.5
Journal of Arthroplasty	169	3	5.8	3.4
Journal of Hand Surgery‑American Volume	144	3	14.8	2.1
Journal of the American Academy of Orthopaedic Surgeons	123	2	8.8	2.6

*CY*, citations per year.

**Table IV:** Institutions for top 100 most-cited total elbow arthroplasty articles

Institutions	Publications	Total citations	Rank list
Mayo Clinic	30	2,800	3, 4, 5, 7, 9, 18, 19, 21, 22, 23, 24, 28, 31, 39, 40, 42, 43, 44, 49, 52, 55, 70, 72, 75, 81, 88, 89, 90, 97, 99
Brigham and Women's Hospital	5	272	25, 53, 85, 92, 95
Hospital for Special Surgery	4	279	27, 34, 38, 83
Amphia Hospital	3	210	13, 50, 96
Sagamihara National Hospital	3	193	29, 45, 78
Harvard Medical School	3	163	30, 85, 92
Leiden University Medical Center	3	156	51, 64, 77
University of Florida	3	138	54, 82, 99
St. Michael's Hospital and University of Toronto	2	328	2, 94
Nuffield Orthopaedic Centre	2	256	11, 14

**Table V:** Top 10 authors for top 100 most-cited total elbow arthroplasty articles

Author	Publications	Total citations	Rank list	Current institution
Morrey, Bernard	35	3,125	3, 4, 5, 7, 9, 17, 18, 19, 21, 22, 23, 24, 28, 31, 39, 40, 42, 43, 44, 46, 49, 52, 55, 59, 65, 66, 68, 70, 72, 81, 88, 89, 90, 97, 99	Department of Orthopedic Surgery, Mayo Clinic, Rochester, MN, USA.
Adams, Robert	10	697	17, 19, 22, 31, 40, 46, 66, 68, 81, 97	Department of Orthopedic Surgery, Mayo Clinic, Rochester, MN, USA.
Sanchez-Sotelo, Joaquin	9	513	21, 39, 42, 44, 55, 72, 88, 97, 99	Department of Orthopedic Surgery, Mayo Clinic, Rochester, MN, USA.
O'Driscoll, Shawn W.	6	381	28, 37, 42, 52, 62, 75	Department of Orthopedic Surgery, Mayo Clinic, Rochester, MN, USA.
Bryan, Richard S.	5	732	5, 7, 18, 23, 49	Department of Orthopedic Surgery, Mayo Clinic, Rochester, MN, USA.
King, Graham J W	5	398	8, 31, 37, 62, 93	Department of Surgery, St. Joseph's Health Care, Hand and Upper Limb Centre, Western University, London, ON, Canada
Schemitsch, Emil H.	4	408	2, 91, 94, 95	Division of Orthopaedic Surgery, Department of Surgery, University of Western Ontario, London, Ontario, Canada.
Figgie, Mark P.	4	286	27, 34, 38, 61	Hospital for Special Surgery, New York, NY.
Inglis, Allan E.	4	286	27, 34, 38, 61	Beth Israel Medical Center, New York, New York, USA.
Stanley, David	4	283	10, 58, 76, 98	Sheffield Teaching Hospitals NHS Trust, Northern General Hospital, Sheffield, UK.

**Table VI:** Senior authors with at least 2 top 100 most-cited total elbow arthroplasty articles

Senior author	Publications	Total citations	Rank list	Institution
Morrey, Bernard	29	2,516	3, 4, 7, 9, 17, 19, 21, 22, 24, 28, 31, 40, 42, 43, 44, 46, 52, 55, 59, 65, 66, 68, 70, 72, 81, 88, 89, 90, 97	Department of Orthopedic Surgery, Mayo Clinic, Rochester, MN, USA.
Stanley, David	4	283	10, 58, 76, 98	Sheffield Teaching Hospitals NHS Trust, Northern General Hospital, Sheffield, UK.
Bryan, Richard S.	3	261	18, 23, 49	Department of Orthopedic Surgery, Mayo Clinic, Rochester, MN, USA.
Rozing, Piet	3	156	51, 64, 77	Department of Orthopaedics, Leiden University Medical Center, Leiden, The Netherlands
Carr, Andrew J.	2	256	11, 14	Nuffield Department of Orthopaedics, Rheumatology and Musculoskeletal Sciences, University of Oxford, Oxford, UK.
King, Graham J W	2	197	8, 93	Department of Surgery, St. Joseph's Health Care, Hand and Upper Limb Centre, Western University, London, ON, Canada
Furnes, Ove	2	157	12, 84	Department of Orthopaedic Surgery, Norwegian Arthroplasty Register, Haukeland University Hospital, Bergen, Norway
Figgie, Harry E.	2	145	34, 38	Department of Orthopaedics, University Hospitals and Case Western Reserve University School of Medicine, Cleveland, Ohio.
Ranawat, Chitranjan S.	2	134	27, 83	Hospital for Special Surgery, New York
Nishino, Junki	2	110	45, 78	Section of Orthopaedics, Sagamihara National Hospital, Sagamihara City, Kanagawa Prefecture, Japan.
Eygendaal, Denise	2	98	50, 96	Department of Orthopaedics and Sports Medicine, Erasmus University Medical Center Rotterdam, Rotterdam, the Netherlands.
Thornhill, Thomas S.	2	97	53, 95	Brigham and Women's Hospital, Boston, MA, USA.
Chen, Neal	2	82	85, 92	Mass General Hospital, Boston, MA, USA.
Schemitsch, Emil H.	2	80	91, 94	Division of Orthopaedic Surgery, Western University, London, ON, Canada.

**Table VII:** Top cited total elbow arthroplasty articles in the last 5 years

Rank	Article	Year	Total citations	Citations per year
1	Total Elbow Arthroplasty: A Systematic Review.	2017	112	14
2	Total elbow replacement: outcome of 1,146 arthroplasties from the Scottish Arthroplasty Project	2013	91	7.6
3	Implant survival after total elbow arthroplasty: a retrospective study of 324 procedures performed from 1980 to 2008	2014	72	6.5
4	Total Elbow Arthroplasty for Distal Humeral Fractures A Ten-Year-Minimum Follow-up Study	2017	67	8.4
5	Why does total elbow arthroplasty fail today? A systematic review of recent literature	2017	61	7.6
6	Primary Linked Semiconstrained Total Elbow Arthroplasty for Rheumatoid Arthritis A Single-Institution Experience with 461 Elbows Over Three Decades	2016	55	6.1
7	Open Reduction and Internal Fixation Versus Total Elbow Arthroplasty for the Treatment of Geriatric Distal Humerus Fractures: A Systematic Review and Meta-Analysis	2014	55	5
8	Total elbow arthroplasty for acute distal humeral fractures in patients over 65 yr old - Results of a multicenter study in 87 patients	2013	55	4.6
9	Complications and revision rate compared by type of total elbow arthroplasty	2013	53	4.4
10	Experience with the Coonrad Morrey total elbow arthroplasty: 78 consecutive total elbow arthroplasties reviewed with an average 5 yr of follow-up	2013	49	4.1

**Figure 4:**
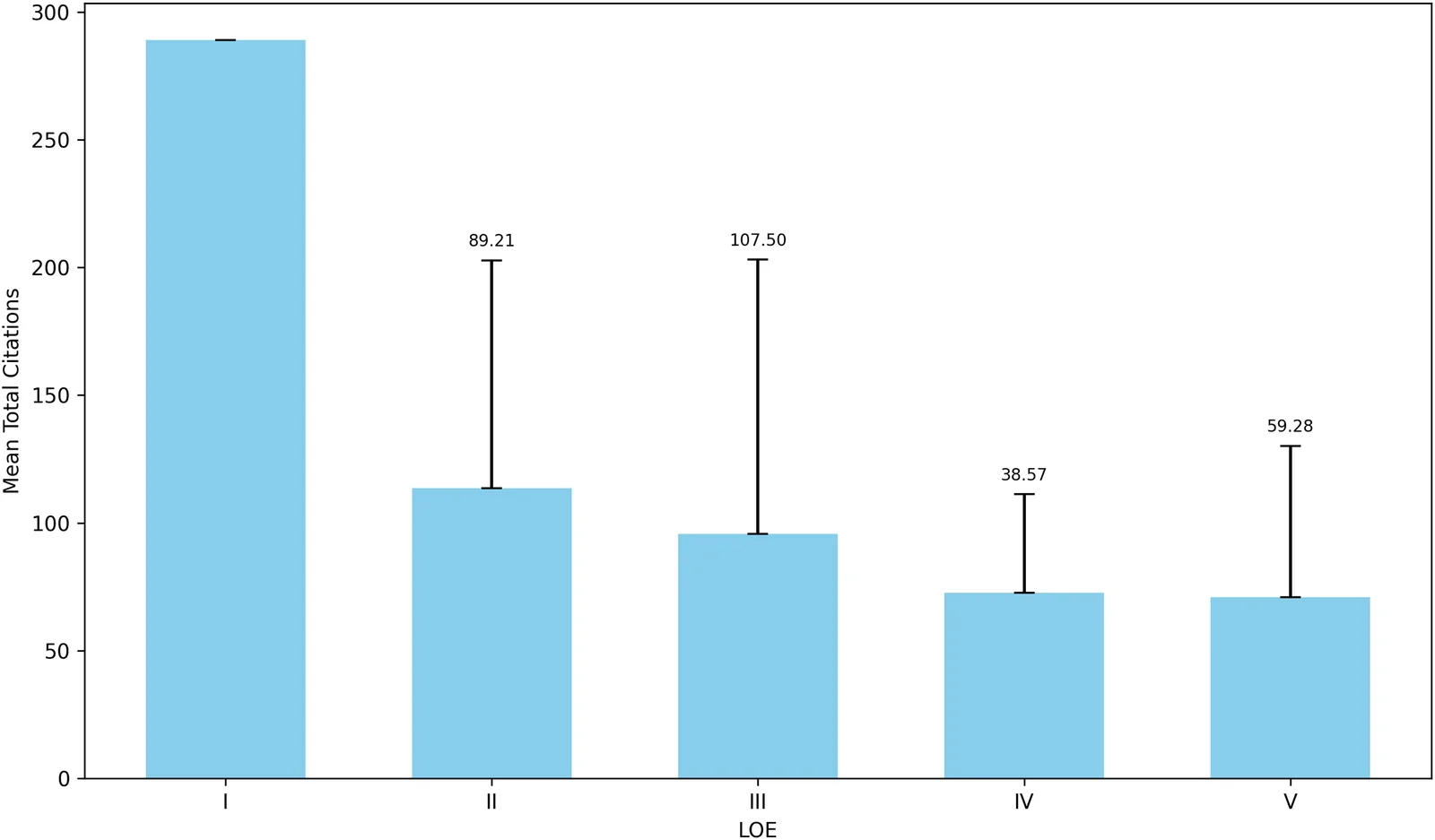
Citations by level of evidence for top 100 most-cited total elbow arthroplasty articles.

A total of 98 clinical studies were found in the most cited TEA articles. Of these, most were level 4 evidence (n = 59, 73 ± 39 citations), followed by level 3 evidence (n = 24, 96 ± 107 citations), level 5 evidence (n = 9, 71 ± 59 citations), level 2 (n = 5, 114 ± 89 citations), and level 1 (n = 1, 289 citations) (Fig. 4). Spearman coefficient analysis demonstrated no association between the level of evidence and number of citations (coefficient = 0.12, *P* = .21). Of the top 100 cited TEA articles, most were retrospective case series (n = 64), followed by comparative case series (n = 11), systematic reviews (n = 6), expert-opinion reviews (n = 6), prospective case series (n = 5), technical notes (n = 3), 2 for each cadaveric studies and case reports (n = 2), and one randomized controlled trial (n = 1).

**Figure 5:**
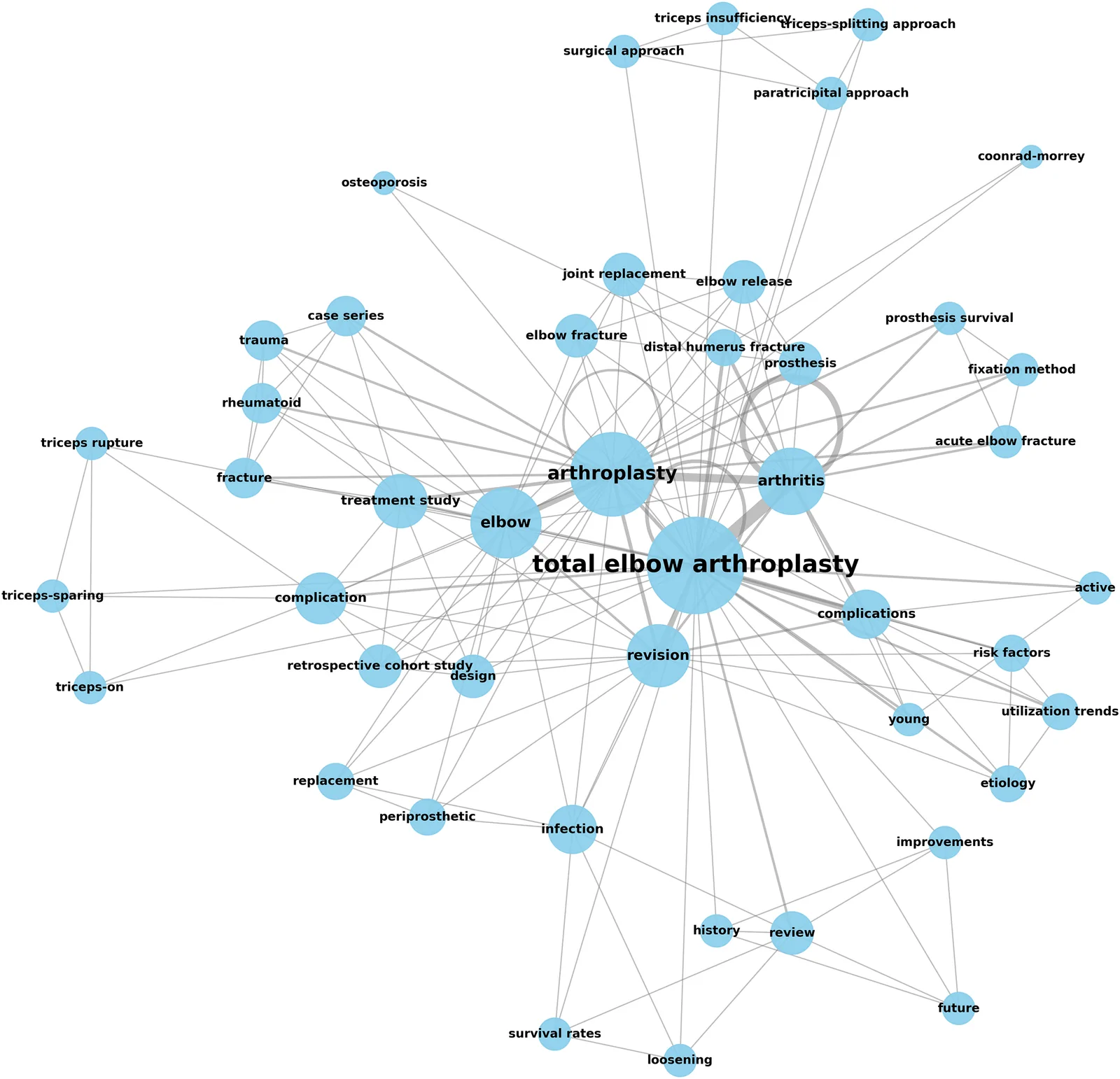
Network plot of keywords for top 100 most-cited total elbow arthroplasty articles.

Keywords were found for 15 articles in the top 100 most-cited TEA articles. Network analysis of these keywords showed a high degree of centrality for TEA, elbow, arthroplasty, arthritis, and revision (Fig. 5).

## Discussion

TEA has evolved into a definitive surgical treatment for patients with severe elbow pathology, particularly those with rheumatoid arthritis, end-stage osteoarthritis, and distal humerus fractures. Over the past several decades, TEA has transitioned from a niche procedure to a well-established intervention, supported by advancements in implant design, surgical technique, and patient selection. While the procedure remains less common than lower extremity arthroplasties, it has demonstrated reliable improvements in pain relief, range of motion, and quality of life in appropriately selected patients. Linked and unlinked prosthetic options have expanded its utility, although the complications (ranging from loosening and triceps insufficiency to periprosthetic fracture and infection) highlight the importance of continued innovation including implant design and surgical technique and well-designed clinical studies for patient outcomes. As the indications continue to broaden, TEA will likely remain a critical part of the treatment armamentarium for upper extremity reconstructive surgery.

This bibliometric analysis examined the 100 most-cited articles on TEA to better understand the research trends, publication impact, and evolution of clinical knowledge surrounding TEA. With 8,225 total citations and an average of 82 citations per article, the findings highlight the strong academic interest in TEA. In contrast to bibliometric data on topics such as anterior cruciate ligament reconstruction (29,629 citations) and total shoulder arthroplasty (19,726 citations), TEA represents a comparatively underpublished area, highlighting potentially the still relative early development of this field and the need for continued high-quality published research.^1^^,^^65^ While not commonly reported for bibliometric analysis for elbow literature, the top 50 lateral epicondylitis articles were found to be cited a total of 6,368 times, further reinforcing the relative lower publication volume surrounding elbow pathologies relative to other major joints.^8^

The most influential work based on the number of CPY in our analysis was by Day et al. This study described the national trends and future projections for shoulder and elbow arthroplasty in the United States, using data from 1993 to 2007.^22^ The authors demonstrated a rapid rise in upper extremity arthroplasty volumes and their findings emphasized not only the increased clinical demand but also a notable rise in revision burden and healthcare costs associated with these procedures. This study brought attention to the growing utilization of shoulder and elbow arthroplasty and served as a foundational reference for health policy discussions and resource planning in orthopedic surgery. Studies such as this are often foundational to frame the importance of future studies in elbow arthroplasty literature, which may be a main reason for its high citation per year. Although the Day et al study published in 2015 had projected upper extremity surgeries, including TEA, to increase by 322%, TEA has remained less prevalent than originally projected since that study. McKissack et al found that the number of TEAs has decreased from 2010 to 2018 by 36.9%.^44^ A more recent study by Ragland et al using publicly available data from 2009 to 2019 for the CMS Medicare Part-B National Summary found TEA growth to be still far lower than what is projected for total knee arthroplasty and total hip arthroplasty.^54^ The projected annual growth rates from 2020 to 2060 for primary and revision TEAs were 1.03% and 5.17%, respectively. Rather than indicating uniform contemporary expansion of TEA, these recent utilization studies suggest a more nuanced pattern. In the United States, primary TEA volume decreased over parts of the 2010s, while indications shifted away from inflammatory arthritis and toward fracture-related and post-traumatic pathology. Although future utilization is projected to increase, the anticipated growth remains modest in absolute terms compared with larger joint arthroplasty. Accordingly, the Day et al study should be interpreted as an important early upper-extremity arthroplasty projection rather than evidence of sustained contemporary growth in TEA specifically.

The second most cited article in our analysis was by McKee et al, which is a multicenter randomized controlled trial comparing TEA and ORIF in elderly patients with displaced intra-articular distal humeral fractures.^43^ The study found that TEA provided superior functional outcomes, more consistent recovery, and a shorter operative time compared to ORIF. It also highlighted a significant portion of fractures initially treated with ORIF that required intraoperative conversion to TEA due to comminution, suggesting limited feasibility of fixation in this population. Studies that involve sentinel work that redefines the clinical treatment paradigm around treatment of an injury, such as the work by McKee et al, are often cited as subsequent studies evolve that either validate or refine the knowledge in that new field. Since the work of McKee et al, multiple subsequent studies have ensured that has supported the use of TEA in complex geriatric distal humerus fractures.^32^^,^^43^^,^^51^^,^^62^

The third most cited article by Gill et al reports long-term outcomes of the Coonrad Morrey TEA in patients with rheumatoid arthritis, with follow-up ranging from 10-15 years.^28^ The study showed high prosthesis survival, excellent pain relief, and meaningful improvements in range of motion and overall function. Despite a moderate complication rate, the vast majority of patients were satisfied with the procedure, and revision surgeries were relatively uncommon. As one of the earliest and most comprehensive long-term evaluations of TEA, this work was instrumental in validating the procedure's durability and effectiveness in managing advanced inflammatory joint disease, which likely contributed to its high citation index.

Interestingly, our bibliometric analysis demonstrated there was a decrease in citation density for the top 100 most-cited TEA articles as publication year increased until the present. This may be attributable to the importance of the early articles in laying the foundation for our understanding of TEA today. In terms of the most cited journals for TEA, our analysis found the top journals as JBJS–American Volume, Journal of Shoulder and Elbow Surgery, and JBJS–British Volume. Other bibliometric analysis on elbow has found similarly JBJS-American Volume to be among the top cited journals; however, they also commonly include American Journal of Sports Medicine, which was absent from our analysis.^8^^,^^64^ After adjusting for the level of evidence and study design, we found no significant correlation between journal impact factor and citation count, suggesting that the influence or popularity of a journal does not strongly determine how frequently a top TEA article is cited or read within the academic community.

Dr. Bernard Morrey contributed to the most number of top-cited TEA articles with 35 articles (rank: 3, 4, 5, 7, 9, 17, 18, 19, 21, 22, 23, 24, 28, 31, 39, 40, 42, 43, 44, 46, 49, 52, 55, 59, 65, 66, 68, 70, 72, 81, 88, 89, 90, 97, and 99) and had 3,125 citations.^5^, ^6^, ^7^^,^^9^, ^10^, ^11^, ^12^^,^^15^^,^^17^^,^^19^^,^^28^^,^^33^, ^34^, ^35^, ^36^, ^37^, ^38^^,^^40^^,^^41^^,^^45^, ^46^, ^47^, ^48^, ^49^, ^50^^,^^55^^,^^57^, ^58^, ^59^, ^60^, ^61^^,^^63^^,^^66^^,^^68^^,^^69^ He pioneered many of today's core elbow arthroplasty principles, including the anterior humeral flange and “sloppy-hinge” concept that reduce loosening. His posterior triceps-preserving exposure likewise remains standard worldwide. The author with the second most publications in the top 100 list was Dr. Robert Adams, who is co-author with Dr. Morrey, with 10 publications (rank: 17, 19, 22, 31, 40, 46, 66, 68, 81, and 97) and 697 citations.^5^^,^^10^^,^^12^^,^^36^, ^37^, ^38^^,^^40^^,^^55^^,^^68^^,^^69^ In terms of senior authorship, Dr. Bernard Morrey had the most at 29 publications.^5^^,^^6^^,^^9^, ^10^, ^11^, ^12^^,^^15^^,^^17^^,^^19^^,^^28^^,^^33^, ^34^, ^35^, ^36^, ^37^, ^38^^,^^40^^,^^41^^,^^49^^,^^50^^,^^55^^,^^57^, ^58^, ^59^^,^^61^^,^^63^^,^^66^^,^^68^^,^^69^ The next senior author is Dr. David Stanley with 4 publications (rank: 10, 58, 76, and 98) and a total of 283 citations.^2^^,^^26^^,^^52^^,^^53^ The majority of articles were from the United States (n = 58) followed by the United Kingdom (n = 12) and the Netherlands (n = 6). The predominance of US publications in our dataset should be interpreted with caution. In part, this likely reflects the historical influence of major US centers, particularly the Mayo Clinic, and the widespread impact of the Coonrad Morrey semi-constrained prosthesis on modern TEA practice and scholarship. However, this finding may also be influenced by citation database structure and language bias, which can preferentially amplify English-language publications. In addition, our geographic analysis was based on absolute publication counts and was not normalized to population size, surgeon workforce, or national research output; therefore, these data should not be interpreted as a measure of per-capita scholarly productivity.

A majority of the top 100 TEA articles were level of evidence 4 (59%). While the argument could be made that higher level of evidence would be cited more frequently, our analysis did not find this correlation (*P* = .21) and likely due to the limited sample size of the higher level of evidence articles in the TEA literature. Of the studies that were included in this bibliometric analysis, there were only 5 level 2 and one level 1 articles. The level 1 article was the randomized control trial by McKee et al that compared the use of TEA with ORIF in elderly patients with displaced intra-articular distal humerus fractures.^43^ The lack of higher level of evidence such as the one by McKee et al may be due to the relative small number of patients that undergo TEA based on current clinical indications. When the pathology or surgical procedure is relatively uncommon, it may be difficult for high-level studies to be completed by single institutions. More pragmatic study designs are therefore more often used for such instances vs. multicenter trials, which may require significant funding resources.

The top cited systematic review for TEA was published in 2017 systematic review by Welsink et al and pooled 73 studies comprising 9,379 TEAs across 19 implant designs, most commonly Souter-Strathclyde, Coonrad Morrey, Kudo, and GSB III.^67^ Overall functional results were good—mean flexion 129°/extension lag 30°—and weighted mean survival was 85.5% for linked and 74% for unlinked prostheses, with complication rates of 11%-38% (clinical loosening 7%). Although performance differs only modestly between designs, this study shows that TEA survivorship and complication profiles still lag behind arthroplasty outcomes in larger joints like the hip and knee. The top cited cadaveric study by O'Driscoll et al measured 3-dimensional motion before and after implantation of a modified Coonrad “loose-hinge” prosthesis in 11 cadaver specimens and found that physiologic varus-valgus laxity rose only modestly, from 2.7° ± 1.5° natively to 3.8° ± 1.4° postimplant, which is far below the device's mechanical limits.^50^ The data confirm that, in vivo, such implants function as semi-constrained systems, with surrounding muscles absorbing forces that would otherwise be transmitted to the bone–prosthesis interface.

While our present study was extensive in terms of the volume of TEA literature evaluated, there are a few limitations worth noting. One limitation is the time-dependent nature of this analysis. It takes time for published articles to be cited, as authors referencing an article needs time to conduct and publish their own study as well. To counteract this potential bias, we used CPY as a secondary ranking metric for articles after total citations. That means that if 2 articles had the same number of citations, then the article that had a higher CPY was ranked above the one that took more to accrue the same number of citations. This can be seen in effect with the rank 17 and rank 18 articles, which both have 103 citations; however, Yamaguchi et al had a CPY of 3.8, while Morrey et al had a CPY of 2.5 (Appendix 1).^46^^,^^68^ Additionally, the outcome measure for the multivariable linear regression on journal impact factor used CPY over total citations. Another potential limitation of the present study is the limitation of using a single database for this bibliometric analysis. While multiple databases (Scopus, Google Scholar, and MEDLINE) all have their own form of citation record keeping, there are inherent discrepancies between them due to which resources and journals are kept in these databases. We therefore chose a single database for quantifying citation numbers. While considering other databases, we ultimately ended up with Web of Science due to their comprehensive breadth of articles, and the usage of various databases alongside their core collection. Furthermore, while citation counts provide an indication of an article's impact, they may not completely reflect the methodological quality or clinical relevance of the studies. Another variable to consider around the use of citations is the potential for authors to self-cite and the potential for a “snowball effect,” where frequently cited articles attract more references over time, leading to the probability of self-selection bias. In addition, there is the potential for language barriers and regional biases toward non-English articles, leading to the potential for under-representing the global understanding and literature of TEA at large. We attempted to mitigate this by using all the databases under Web of Science, which include the Chinese Science Citation Database, Russian Science Citation Index, and the Arabic Citation Index. Finally, although this approach improved precision for TEA-focused articles, it may have reduced sensitivity for studies indexed under alternative terminology such as “total elbow replacement,” “TEA,” or implant-specific descriptors. Accordingly, some relevant articles may not have been captured.

## Conclusion

TEA has progressed from a niche intervention into a well-established solution for complex elbow pathology, with ongoing refinements in surgical technique, implant design, and patient selection shaping its modern role. This bibliometric analysis of the 100 most-cited TEA articles highlights a steady academic interest, although citation volumes remain lower than those seen in other major arthroplasty or orthopedic domains. Foundational work by Day et al, McKee et al, and Gill et al reflects a shift in clinical practice, toward broader adoption of TEA in fracture care and inflammatory arthritis, while also underscoring the importance of long-term outcome studies in validating surgical innovation. Although most TEA studies are categorized as level 4 evidence—largely because the small patient pool makes randomized controlled trials impractical—citation analyses reveal that clinical impact and real-world relevance often attract more academic attention than study design alone. As the population ages and TEA case volumes grow, however, there will be greater opportunity and need to conduct robust, prospective trials that elevate the evidence base. Together, these findings emphasize the field's evolution and continued need for robust, prospective research to inform best practices and optimize patient outcomes in TEA.

## Disclaimers:

Funding: No funding was disclosed by the authors.

Conflicts of interest: Kevin A. Hao reports that he is a paid consultant for LinkBio Corp.

Xinning Li reports that he is a paid consultant and receives royalties from FH Ortho.

Richard Ma reports that he is a paid consultant for Johnson & Johnson MedTech.

Robert L. Parisien reports that he is a paid consultant for Arthrex, Inc. and Vericel Corporation.

Josef K. Eichinger reports that he is a paid consultant for Arthrex and Exactech.

Any additional authors, their immediate families, and any research foundations with which they are affiliated have not received any financial payments or other benefits from any commercial entity related to the subject of this article.

## Supplementary Data

TEA Appendix
