# Supplementary material for: The most influential articles in total elbow arthroplasty: bibliometric analysis of the top 100 most-cited articles
Source: JSES Rev Rep Tech. 2026 Jul 17;6(4):100821. doi: 10.1016/j.xrrt.2026.100821 (PMC13543903; doi:10.1016/j.xrrt.2026.100821)
Supplement: Supplementary file 1 [file mmc1.docx]

**Appendix 1: List of Top 100 Most-Cited Total Elbow Arthroplasty Articles**

| **Rank** | **Title** | **Authors** | **Country** | **Year** | **Total Citations** | **CY** |
| --- | --- | --- | --- | --- | --- | --- |
| 1 | Prevalence and projections of total shoulder and elbow arthroplasty in the United States to 2015 | Day, Judd S.; Lau, Edmund; Ong, Kevin L.; Williams, Gerald R.; Ramsey, Matthew L.; Kurtz, Steven M. | USA | 2010 | 530 | 35.3 |
| 2 | A multicenter, prospective, randomized, controlled trial of open reduction-internal fixation versus total elbow arthroplasty for displaced intra-articular distal humeral fractures in elderly patients | Mckee, Michael D.; VeilLette, Christian J. H.; Hall, Jeremy A.; Schemitsch, Emit H.; Wild, Lisa M.; McCormack, Robert; Perey, Bertrand; Goetz, Thomas; Zomar, Mauri; Moon, Karyn; Mande, Scott; Petit, Shirlet; Guy, Pierre; Leung, Irene | Canada | 2009 | 289 | 18.1 |
| 3 | The Coonrad-Morrey total elbow arthroplasty in patients who have rheumatoid arthritis - A ten to fifteen-year follow-up study | Gill, DRJ; Morrey, BF | USA | 1998 | 271 | 10 |
| 4 | Total elbow arthroplasty as primary treatment for distal humeral fractures in elderly patients | Cobb, TK; Morrey, BF | USA | 1997 | 255 | 9.1 |
| 5 | TOTAL ELBOW ARTHROPLASTY - A 5-YEAR EXPERIENCE AT THE MAYO-CLINIC | MORREY, BF; BRYAN, RS; DOBYNS, JH; LINSCHEID, RL | USA | 1981 | 246 | 5.6 |
| 6 | A comparison of open reduction and internal fixation and primary total elbow arthroplasty in the treatment of intraarticular distal humerus fractures in women older than age 65 | Frankle, MA; Herscovici, D; DiPasquale, TG; Vasey, MB; Sanders, RW | USA | 2003 | 228 | 10.4 |
| 7 | EXTENSIVE POSTERIOR EXPOSURE OF THE ELBOW - A TRICEPS-SPARING APPROACH | BRYAN, RS; MORREY, BF | USA | 1982 | 225 | 5.2 |
| 8 | Functional outcome of semiconstrained total elbow arthroplasty | Hildebrand, KA; Patterson, SD; Regan, WD; MacDermid, JC; King, GJW | Canada | 2000 | 158 | 6.3 |
| 9 | Distal humeral fractures treated with noncustom total elbow replacement | Kamineni, S; Morrey, BF | USA | 2004 | 149 | 7.1 |
| 10 | Complex fractures of the distal humerus in the elderly - The role of total elbow replacement as primary treatment | Garcia, JA; Mykula, R; Stanley, D | UK | 2002 | 146 | 6.3 |
| 11 | Total elbow arthroplasty | Little, CP; Graham, AJ; Carr, AJ | UK | 2005 | 145 | 7.2 |
| 12 | Results after 562 total elbow replacements: A report from the Norwegian Arthroplasty Register | Fevang, Bjorg-Tilde S.; Lie, Stein A.; Havelin, Leif I.; Skredderstuen, Arne; Furnes, Ove | Norway | 2009 | 113 | 7.1 |
| 13 | Total Elbow Arthroplasty: A Systematic Review. | Welsink, Chantal L; Lambers, Kaj T A; van Deurzen, Derek F P; Eygendaal, Denise; van den Bekerom, Michel P J | Netherlands | 2017 | 112 | 14 |
| 14 | Outcomes of total elbow arthroplasty for rheumatoid arthritis: Comparative study of three implants | Little, CP; Graham, AJ; Karatzas, G; Woods, DA; Carr, AJ | UK | 2005 | 111 | 5.6 |
| 15 | Total elbow arthroplasty with the Coonrad/Coonrad-Morrey prosthesis - A 10-to 31-year survival analysis | Aldridge, JM; Lightdale, NR; Mallon, WJ; Coonrad, RW | USA | 2006 | 110 | 5.8 |
| 16 | Indications and Reoperation Rates for Total Elbow Arthroplasty: An Analysis of Trends in New York State | Gay, David M.; Lyman, Stephen; Huong Do; Hotchkiss, Robert N.; Marx, Robert G.; Daluiski, Aaron | USA | 2012 | 106 | 8.2 |
| 17 | Infection after total elbow arthroplasty | Yamaguchi, K; Adams, RA; Morrey, BF | USA | 1998 | 103 | 3.8 |
| 18 | INFECTION AFTER TOTAL ELBOW ARTHROPLASTY | MORREY, BF; BRYAN, RS | USA | 1983 | 103 | 2.5 |
| 19 | Instability of the elbow treated with semiconstrained total elbow arthroplasty | Ramsey, ML; Adams, RA; Morrey, BF | USA | 1999 | 102 | 3.9 |
| 20 | Total elbow arthroplasty as primary treatment for distal humeral fractures in elderly patients | Ray, PS; Kakarlapudi, K; Rajsekhar, C; Bhamra, MS | UK | 2000 | 99 | 4 |
| 21 | Linked elbow replacement: A salvage procedure for distal humeral nonunion | Cil, Akin; Veillette, Christian J. H.; Sanchez-Sotelo, Joaquin; Morrey, Bernard F. | USA | 2008 | 98 | 5.8 |
| 22 | Polyethylene wear after total elbow arthroplasty | Lee, BP; Adams, RA; Morrey, BF | USA | 2005 | 98 | 4.9 |
| 23 | COMPLICATIONS OF TOTAL ELBOW ARTHROPLASTY | MORREY, BF; BRYAN, RS | USA | 1982 | 96 | 2.2 |
| 24 | Total Elbow Arthroplasty in Patients Forty Years of Age or Less | Celli, Andrea; Morrey, Bernard F. | USA | 2009 | 93 | 5.8 |
| 25 | CAPITELLOCONDYLAR TOTAL ELBOW ARTHROPLASTY - 2-YEAR TO 5-YEAR FOLLOW-UP IN RHEUMATOID-ARTHRITIS | EWALD, FC; SCHEINBERG, RD; POSS, R; THOMAS, WH; SCOTT, RD; SLEDGE, CB | USA | 1980 | 93 | 2.1 |
| 26 | Total elbow replacement: outcome of 1,146 arthroplasties from the Scottish Arthroplasty Project | Jenkins, Paul J.; Watts, Adam C.; Norwood, Tim; Duckworth, Andrew D.; Rymaszewski, Lech A.; McEachan, Jane E. | UK | 2013 | 91 | 7.6 |
| 27 | PRIMARY SEMICONSTRAINED TOTAL ELBOW ARTHROPLASTY - SURVIVAL ANALYSIS OF 113 CONSECUTIVE CASES | KRAAY, MJ; FIGGIE, MP; INGLIS, AE; WOLFE, SW; RANAWAT, CS | USA | 1994 | 89 | 2.9 |
| 28 | KINEMATICS OF SEMI-CONSTRAINED TOTAL ELBOW ARTHROPLASTY | ODRISCOLL, SW; AN, KN; KORINEK, S; MORREY, BF | USA | 1992 | 89 | 2.7 |
| 29 | TOTAL ELBOW ARTHROPLASTY WITH A NONCONSTRAINED SURFACE-REPLACEMENT PROSTHESIS IN PATIENTS WHO HAVE RHEUMATOID-ARTHRITIS - A LONG-TERM FOLLOW-UP-STUDY | KUDO, H; IWANO, K | Japan | 1990 | 83 | 2.4 |
| 30 | Complications of Total Elbow Arthroplasty | Kim, Jaehon M.; Mudgal, Chaitanya S.; Konopka, Joseph F.; Jupiter, Jesse B. | USA | 2011 | 81 | 5.8 |
| 31 | Total elbow arthroplasty: Revision with use of a non-custom semiconstrained prosthesis | King, GJW; Adams, RA; Morrey, BF | USA | 1997 | 81 | 2.9 |
| 32 | Total elbow arthroplasty in rheumatoid arthritis A population-based study from the Finnish Arthroplasty Register | Skytta, Eerik T.; Eskelinen, Antti; Paavolainen, Pekka; Ikavalko, Mikko; Remes, Ville | Finland | 2009 | 79 | 4.9 |
| 33 | Survivorship and radiological analysis of the standard Souter-Strathclyde total elbow arthroplasty | Trail, IA; Nuttall, D; Stanley, JK | UK | 1999 | 78 | 3 |
| 34 | SALVAGE OF NON-UNION OF SUPRACONDYLAR FRACTURE OF THE HUMERUS BY TOTAL ELBOW ARTHROPLASTY | FIGGIE, MP; INGLIS, AE; MOW, CS; FIGGIE, HE | USA | 1989 | 76 | 2.1 |
| 35 | Total elbow arthroplasty for the treatment of insufficient distal humeral fractures. A retrospective clinical study and review of the literature | Chalidis, B.; Dimitriou, C.; Papadopoulos, P.; Petsatodis, G.; Giannoudis, P. V. | UK | 2009 | 73 | 4.6 |
| 36 | Implant survival after total elbow arthroplasty: a retrospective study of 324 procedures performed from 1980 to 2008 | Plaschke, Hans Christian; Thillemann, Theis M.; Brorson, Stig; Olsen, Bo S. | Denmark | 2014 | 72 | 6.5 |
| 37 | Modes of wear after semicionstrained total elbow arthroplasty | Goldberg, Steven H.; Urban, Robert M.; Jacobs, Joshua J.; King, Graham J. W.; O'Driscoll, Shawn W.; Cohen, Mark S. | USA | 2008 | 69 | 4.1 |
| 38 | TOTAL ELBOW ARTHROPLASTY FOR COMPLETE ANKYLOSIS OF THE ELBOW | FIGGIE, MP; INGLIS, AE; MOW, CS; FIGGIE, HE | USA | 1989 | 69 | 1.9 |
| 39 | Total Elbow Arthroplasty for Distal Humeral Fractures A Ten-Year-Minimum Follow-up Study | Barco, Raul; Streubel, Philipp N.; Morrey, Bernard F.; Sanchez-Sotelo, Joaquin | Spain | 2017 | 67 | 8.4 |
| 40 | Triceps insufficiency following total elbow arthroplasty | Celli, A; Arash, A; Adams, RA; Morrey, BF | USA | 2005 | 67 | 3.4 |
| 41 | TOTAL ELBOW ARTHROPLASTY - AN 18-YEAR EXPERIENCE | KASTEN, MD; SKINNER, HB | USA | 1993 | 67 | 2.1 |
| 42 | Periprosthetic humeral fractures after total elbow arthroplasty: Treatment with implant revision and strut allograft augmentation | Sanchez-Sotelo, J; O'Driscoll, S; Morrey, BF | USA | 2002 | 66 | 2.9 |
| 43 | Semiconstrained total elbow arthroplasty for ankylosed and stiff elbows | Mansat, P; Morrey, BF | USA | 2000 | 66 | 2.6 |
| 44 | Failure Patterns After Linked Semiconstrained Total Elbow Arthroplasty for Posttraumatic Arthritis | Throckmorton, Thomas; Zarkadas, Peter; Sanchez-Sotelo, Joaquin; Morrey, Bernard | USA | 2010 | 65 | 4.3 |
| 45 | Total elbow arthroplasty with use of a nonconstrained humeral component inserted without cement in patients who have rheumatoid arthritis | Kudo, H; Iwano, K; Nishino, J | Japan | 1999 | 64 | 2.5 |
| 46 | Total elbow arthroplasty in primary osteoarthritis of the elbow | Kozak, TKW; Adams, RA; Morrey, BF | USA | 1998 | 64 | 2.4 |
| 47 | Complication and Revision Rates Following Total Elbow Arthroplasty | Krenek, Lucie; Farng, Eugene; Zingmond, David; SooHoo, Nelson F. | USA | 2011 | 62 | 4.4 |
| 48 | The triceps preserving approach to total elbow arthroplasty | Pierce, TD; Herndon, HJ | USA | 1998 | 62 | 2.3 |
| 49 | REVISION TOTAL ELBOW ARTHROPLASTY | MORREY, BF; BRYAN, RS | USA | 1987 | 62 | 1.6 |
| 50 | Why does total elbow arthroplasty fail today? A systematic review of recent literature | Prkic, Ante; Welsink, Chantal; The, Bertram; van den Bekerom, Michel P. J.; Eygendaal, Denise | Netherlands | 2017 | 61 | 7.6 |
| 51 | Systematic review of primary total elbow prostheses used for the rheumatoid elbow | van der Lugt, JCT; Rozing, PM | Netherlands | 2004 | 59 | 2.8 |
| 52 | LOOSE-HINGE TOTAL ELBOW ARTHROPLASTY - AN EXPERIMENTAL-STUDY OF THE EFFECTS OF IMPLANT ALIGNMENT ON 3-DIMENSIONAL ELBOW KINEMATICS | SCHUIND, F; ODRISCOLL, S; KORINEK, S; AN, KN; MORREY, BF | USA | 1995 | 59 | 2 |
| 53 | Semiconstrained primary and revision total elbow arthroplasty with use of the Coonrad-Morrey prosthesis | Shi, Lewis L.; Zurakowski, David; Jones, Deryk G.; Koris, Mark J.; Thornhill, Thomas S. | USA | 2007 | 58 | 3.2 |
| 54 | Total elbow arthroplasty failure due to overuse, C-ring failure, and/or bushing wear | Wright, TW; Hastings, H | USA | 2005 | 58 | 2.9 |
| 55 | Primary Linked Semiconstrained Total Elbow Arthroplasty for Rheumatoid Arthritis A Single-Institution Experience with 461 Elbows Over Three Decades | Sanchez-Sotelo, Joaquin; Baghdadi, Yaser M. K.; Morrey, Bernard F. | USA | 2016 | 55 | 6.1 |
| 56 | Open Reduction and Internal Fixation Versus Total Elbow Arthroplasty for the Treatment of Geriatric Distal Humerus Fractures: A Systematic Review and Meta-Analysis | Githens, Michael; Yao, Jeffrey; Sox, Alex H. S.; Bishop, Julius | Stanford | 2014 | 55 | 5 |
| 57 | Total elbow arthroplasty for acute distal humeral fractures in patients over 65 years old - Results of a multicenter study in 87 patients | Mansat, P.; Degorce, H. Nouaille; Bonnevialle, N.; Demezon, H.; Fabre, T. | France | 2013 | 55 | 4.6 |
| 58 | Total elbow arthroplasty for distal humeral fractures: indications, surgical approach, technical tips, and outcome. | Ali, Amjid; Shahane, Shantanu; Stanley, David | UK | 2010 | 55 | 3.7 |
| 59 | Distal humeral fractures treated with noncustom total elbow replacement. Surgical technique. | Kamineni, S; Morrey, Bernard F | UK | 2005 | 54 | 2.7 |
| 60 | Complications and revision rate compared by type of total elbow arthroplasty | Park, Sang-Eun; Kim, Jung-Youn; Cho, Sung-Wook; Rhee, Seung-Koo; Kwon, Soon-Yong | South Korea | 2013 | 53 | 4.4 |
| 61 | TOTAL ELBOW ARTHROPLASTY | GOLDBERG, VM; FIGGIE, HE; INGLIS, AE; FIGGIE, MP | USA | 1988 | 52 | 1.4 |
| 62 | Elbow arthroplasty using a convertible implant. | Gramstad, Gregory D; King, Graham J W; O'Driscoll, Shawn W; Yamaguchi, Ken | USA | 2005 | 51 | 2.6 |
| 63 | Comprehensive assessment of clinical outcome and quality of life after total elbow arthroplasty | Angst, F; John, M; Pap, G; Mannion, AF; Herren, DB; Flury, M; Aeschlimann, A; Schwyzer, HK; Simmen, BR | Switzerland | 2005 | 51 | 2.6 |
| 64 | Primary Souter-Strathclyde total elbow prosthesis in rheumatoid arthritis | van der Lugt, JCT; Geskus, RB; Rozing, PM | Netherlands | 2004 | 51 | 2.4 |
| 65 | Total elbow arthroplasty in patients who have juvenile rheumatoid arthritis | Connor, PM; Morrey, BF | USA | 1998 | 51 | 1.9 |
| 66 | Reimplantation of a total elbow prosthesis following resection arthroplasty for infection | Cheung, Emilie V.; Adams, Robert A.; Morrey, Bernard F. | Stanford | 2008 | 50 | 2.9 |
| 67 | Results of total elbow arthroplasty in the treatment of distal humerus fractures in elderly Asian patients | Lee, Keng Thiam; Lai, Choon Hin; Singh, Sarbjit | Singapore | 2006 | 50 | 2.6 |
| 68 | Coonrad-Morrey total elbow arthroplasty for tumours of the distal humerus and elbow | Athwal, GS; Chin, PY; Adams, RA; Morrey, BF | Canada | 2005 | 50 | 2.5 |
| 69 | Experience with the Coonrad-Morrey total elbow arthroplasty: 78 consecutive total elbow arthroplasties reviewed with an average 5 years of follow-up | Mansat, Pierre; Bonnevialle, Nicolas; Rongieres, Michel; Mansat, Michel; Bonnevialle, Paul | France | 2013 | 49 | 4.1 |
| 70 | Revision total elbow arthroplasty for prosthetic fractures | Athwal, George S.; Morrey, Bernard F. | USA | 2006 | 49 | 2.6 |
| 71 | Kudo total elbow arthroplasty in patients with rheumatoid arthritis - A long-term follow-up study | Tanaka, N; Kudo, H; Iwano, K; Sakahashi, H; Sato, E; Ishii, S | Japan | 2001 | 49 | 2 |
| 72 | Allograft-Prosthetic Composite Reconstruction for Massive Bone Loss Including Catastrophic Failure in Total Elbow Arthroplasty | Morrey, Mark E.; Sanchez-Sotelo, Joaquin; Abdel, Matthew P.; Morrey, Bernard F. | USA | 2013 | 48 | 4 |
| 73 | Characteristics and outcome of 27 elbow periprosthetic joint infections: results from a 14-year cohort study of 358 elbow prostheses | Achermann, Y.; Vogt, M.; Spormann, C.; Kolling, C.; Remschmidt, C.; Wuest, J.; Simmen, B.; Trampuz, A. | Switzerland | 2011 | 48 | 3.4 |
| 74 | Five- to thirteen-year follow-up of the GSB III total elbow arthroplasty | Kelly, EW; Coghlan, J; Bell, S | USA | 2004 | 48 | 2.3 |
| 75 | Total elbow prosthesis loosening caused by ulnar component pistoning | Cheung, Emilie V.; O'Driscoll, Shawn W. | USA | 2007 | 47 | 2.6 |
| 76 | Total elbow arthroplasty for non-rheumatoid patients with a fracture of the distal humerus A MINIMUM TEN-YEAR FOLLOW-UP | Prasad, N.; Ali, A.; Stanley, D. | UK | 2016 | 46 | 5.1 |
| 77 | Souter-strathclyde total elbow arthroplasty - A long-term follow-up study | Rozing, P | Netherlands | 2000 | 46 | 1.8 |
| 78 | Cementless or hybrid total elbow arthroplasty with titanium-alloy implants. A study of interim clinical results and specific complications. | Kudo, H; Iwano, K; Nishino, J | Japan | 1994 | 46 | 1.5 |
| 79 | Complex fractures of the distal humerus in the elderly: Is primary total elbow arthroplasty a valid treatment alternative? A series of 20 cases | Ducrot, G.; Ehlinger, M.; Adam, P.; Di Marco, A.; Clavert, P.; Bonnomet, F. | France | 2013 | 45 | 3.8 |
| 80 | Comparison of functional outcomes of total elbow arthroplasty vs plate fixation for distal humerus fractures in osteoporotic elbows. | Egol, Kenneth A; Tsai, Peter; Vazques, Oscar; Tejwani, Nirmal C | USA | 2011 | 45 | 3.2 |
| 81 | Allograft-prosthesis composite for revision of catastrophic failure of total elbow arthroplasty | Mansat, P; Adams, RA; Morrey, BF | USA | 2004 | 45 | 2.1 |
| 82 | Functional outcome comparison of semiconstrained and unconstrained total elbow arthroplasties | Wright, TW; Wong, AM; Jaffe, R | USA | 2000 | 45 | 1.8 |
| 83 | THE OSTEO-ANCONEUS FLAP - AN APPROACH FOR TOTAL ELBOW ARTHROPLASTY | WOLFE, SW; RANAWAT, CS | USA | 1990 | 45 | 1.3 |
| 84 | A survivorship study of 838 total elbow replacements: a report from the Norwegian Arthroplasty Register 1994-2016 | Krukhaug, Yngvar; Hallan, Geir; Dybvik, Eva; Lie, Stein A.; Furnes, Ove N. | Norway | 2018 | 44 | 6.3 |
| 85 | Total Elbow Arthroplasty | Zhang, Dafang; Chen, Neal | USA | 2019 | 43 | 7.2 |
| 86 | Total elbow arthroplasty: outcomes after triceps-detaching and triceps-sparing approaches | Dachs, Robert P.; Fleming, Mark A.; Chivers, David A.; Carrara, Henri R.; Du Plessis, Jean-Pierre; Vrettos, Basil C.; Roche, Stephen J. | South Africa | 2015 | 43 | 4.3 |
| 87 | Unlinked elbow arthroplasty as primary treatment for fractures of the distal humerus | Kologrianitis, S.; Sinopidis, C.; El Meligy, M.; Rawal, A.; Frostick, S. P. | UK | 2008 | 43 | 2.5 |
| 88 | Total Elbow Arthroplasty | Sanchez-Sotelo, Joaquin; Morrey, Bernard F. | USA | 2011 | 42 | 3 |
| 89 | Proximal ulnar reconstruction with strut allograft in revision total elbow arthroplasty | Kamineni, S; Morrey, BF | USA | 2004 | 42 | 2 |
| 90 | Total elbow arthroplasty after resection of tumors at the elbow | Sperling, JW; Pritchard, DJ; Morrey, BF | USA | 1999 | 42 | 1.6 |
| 91 | Internal fixation versus arthroplasty of comminuted fractures of the distal humerus | Obremskey, WT; Bhandari, M; Dirsehl, DR; Shemitsch, E | USA | 2003 | 41 | 1.9 |
| 92 | Risk factors for reoperation after total elbow arthroplasty | Perretta, Donato; van Leeuwen, Wouter F.; Dyer, George; Ring, David; Chen, Neal | USA | 2017 | 39 | 4.9 |
| 93 | The Lateral Para-Olecranon Approach for Total Elbow Arthroplasty | Studer, Alexis; Athwal, George S.; MacDermid, Joy C.; Faber, Kenneth J.; King, Graham J. W. | Canada | 2013 | 39 | 3.2 |
| 94 | Effect of humeral condylar resection on strength and functional outcome after semiconstrained total elbow arthroplasty | McKee, MD; Pugh, DMW; Richards, RR; Pedersen, E; Jones, C; Schemitsch, EH | Canada | 2003 | 39 | 1.8 |
| 95 | Results of total elbow arthroplasty after excision of the radial head and synovectomy in patients who had rheumatoid arthritis | Schemitsch, EH; Ewald, FC; Thornhill, TS | USA | 1996 | 39 | 1.3 |
| 96 | Total elbow arthroplasty is moving forward: Review on past, present and future. | Prkic, Ante; van Bergen, Christiaan Ja; The, Bertram; Eygendaal, Denise | Netherlands | 2016 | 37 | 4.1 |
| 97 | Long-Term Outcome of Resection Arthroplasty for the Failed Total Elbow Arthroplasty | Zarkadas, Peter C.; Cass, Benjamin; Throckmorton, Thomas; Adams, Robert; Sanchez-Sotelo, Joaquin; Morrey, Bernard F. | USA | 2010 | 37 | 2.5 |
| 98 | Two-stage revision for the treatment of the infected total elbow arthroplasty | Peach, C. A.; Nicoletti, S.; Lawrence, T. M.; Stanley, D. | UK | 2013 | 36 | 3 |
| 99 | Total elbow arthroplasty for primary osteoarthritis | Schoch, Bradley S.; Werthel, Jean-David; Sanchez-Sotelo, Joaquin; Morrey, Bernard F.; Morrey, Mark | USA | 2017 | 35 | 4.4 |
| 100 | Outcomes, complications, utilization trends, and risk factors for primary and revision total elbow replacement | Lovy, Andrew J.; Keswani, Aakash; Dowdell, James; Koehler, Steven; Kim, Jaehon; Hausman, Michael R. | USA | 2016 | 34 | 3.8 |
